# Prognostic Markers in Tyrosine Kinases Specific to Basal-like 2 Subtype of Triple-Negative Breast Cancer

**DOI:** 10.3390/ijms25031405

**Published:** 2024-01-24

**Authors:** Praopim Limsakul, Pongsakorn Choochuen, Thawirasm Jungrungrueang, Krit Charupanit

**Affiliations:** 1 Division of Physical Science, Faculty of Science, Prince of Songkla University, Songkhla 90110, Thailand; praopim.l@psu.ac.th; 2 Center of Excellence for Trace Analysis and Biosensor (TAB-CoE), Faculty of Science, Prince of Songkla University, Songkhla 90110, Thailand; 3 Department of Biomedical Sciences and Biomedical Engineering, Faculty of Medicine, Prince of Songkla University, Songkhla 90110, Thailand; pongsakorn.mwit20@gmail.com (P.C.); thawirasm.j@psu.ac.th (T.J.)

**Keywords:** triple-negative breast cancer, basal-like subtype, tyrosine kinase, transcriptomic analysis, prognostic markers, biomarker

## Abstract

Triple-negative breast cancer (TNBC), a heterogeneous and therapeutically challenging subtype, comprises over 50% of patients categorized into basal-like 1 (BL1) and basal-like 2 (BL2) intrinsic molecular subtypes. Despite their shared basal-like classification, BL2 is associated with a poor response to neoadjuvant chemotherapy and reduced relapse-free survival compared to BL1. Here, the study focused on identifying subtype-specific markers for BL2 through transcriptomic analysis of TNBC patients using RNA-seq and clinical integration. Six receptor tyrosine kinase (TK) genes, including *EGFR*, *EPHA4*, *EPHB2*, *PDGFRA*, *PDGFRB*, and *ROR1*, were identified as potential differentiators for BL2. Correlations between TK mRNA expression and TNBC prognosis, particularly *EGFR*, *PDGFRA*, and *PDGFRB*, revealed potential synergistic interactions in pathways related to cell survival and proliferation. Our findings also suggest promising dual markers for predicting disease prognosis. Furthermore, RT-qPCR validation demonstrated that identified BL2-specific TKs were expressed at a higher level in BL2 than in BL1 cell lines, providing insights into unique characteristics. This study advances the understanding of TNBC heterogeneity within the basal-like subtypes, which could lead to novel clinical treatment approaches and the development of targeted therapies.

## 1. Introduction

Triple-negative breast cancer (TNBC) accounts for 10–20% of all breast cancers, the second leading cause of cancer-related deaths in women, with the annual incidence of breast cancers persistently rising [1]. In comparison to other breast cancer subtypes, TNBC is more prevalent in young women and is associated with aggressive characteristics and poor prognosis, including increased recurrence and metastasis rates [2,3,4]. TNBC is characterized by the absence of estrogen receptor (ER), progesterone receptor (PR), and human epidermal growth factor receptor 2 (HER2). These receptors play crucial roles in the growth and progression of breast cancer and are typically used to determine the subtype of breast cancer as well as guide treatment decisions [4]. Due to its heterogeneity and lacking well-defined receptors, TNBC has been categorized into several molecular-subtype classifications, contributing to the development of the precise treatment for TNBC patients.

The advent of bioinformatics analysis further classifies TNBC into four intrinsic molecular subtypes, including basal-like 1 (BL1), basal-like 2 (BL2), mesenchymal (M), and luminal androgen receptor (LAR), each of which has different clinical features and prognoses [5,6]. The BL1 subtype is distinguished by genetic dependencies on cell cycle and DNA repair genes, whereas the BL2 subtype is abundant in growth factor receptors, adhesion-motility genes, and transcription factors. The M subtype is linked to the expression of genes associated with the epithelial-to-mesenchymal transition. The LAR subtype is characterized by the presence of the androgen receptor and luminal gene signature. Among these subtypes, BL2 patients were observed to have the highest risk for disease progression and tend to have the poorest prognosis, with the shortest median survival of 2.4 years [7] and lowest pathological complete response (pCR of 0%) to neoadjuvant chemotherapy, compared to BL1 (52%), and LAR (10%) [2,6,7,8].

Despite similarities in the basal-like subtype, characterized by the expression of genes associated with basal mammary epithelial cells and high proliferation rates, such as high Ki-67 mRNA expression (*MKI67*) and enrichment of proliferation genes, the BL1 and BL2 subtypes exhibit notable differences in histopathological subtypes and clinical features [2,6,8]. Distinctive patterns, such as medullary carcinomas, are associated with BL1, while metaplastic carcinomas are linked to BL2. In addition, the BL1 subtype has a significantly higher proliferation rate; however, the BL2 subtype generally tends to have a worse prognosis and experiences shorter relapse-free survival [6,8]. The increased expression of growth factor genes and adhesion-motility genes in BL2 may contribute to their aggressive behavior. The low pCR rates in BL2 could stem from the difference in gene ontologies involving growth factor signaling, such as the EGF, MET, and IGF-IR pathways [2,6]. Thus, identifying BL2 subtype-specific biomarkers could be beneficial for developing targeted therapy and diagnosis uses.

Protein tyrosine kinases (TKs) serve as promising biomarkers for identifying the molecular subtype of TNBC and act as targets for novel therapeutic strategies [9]. With approximately 90 TKs among over 500 protein kinases in the human genome, TKs play a key role in regulating essential cellular activities [10]. In TNBC, receptor tyrosine kinases (RTKs) are overexpressed, such as the ErbB family (e.g., EGFR (ErbB1) and HER4 (ErbB4)) [11,12,13,14], the Eph receptor family (e.g., EPHA1, EPHA4, EPHA7, EPHB4, and EPHB6) [15], the platelet-derived growth factor receptor (PDGFR) family (e.g., PDGFRα) [16], the vascular endothelial growth factor receptor (VEGFR) family (e.g., VEGFR1, VEGFR2) [15,16], C-Kit [17,18], and PTK7 [19]. Additionally, non-receptor tyrosine kinases (NRTKs) such as SRC family kinases (e.g., SRC and FYN) and immune-related TKs (e.g., ITK and ZAP70) show elevated expression in TNBC [20]. Targeting these kinases using small molecules or antibodies has proven successful for cancer treatment, including breast cancer. In particular, EGFR is a potential target in TNBC due to its frequent overexpression [21]. However, clinical trials targeting EGFR in TNBC have encountered challenges due to cancer genetic heterogeneity [21,22]. Alternatively, the inhibition of multiple tyrosine kinases has shown efficacy in clinical trials for TNBC [23].

While dysregulated TK activity is frequently associated with cancer, making TKs attractive targets for anti-cancer treatments and prognostic markers for therapy response [24], there are currently no successful TK-associated therapies for TNBC, especially for the BL2 subtype. Here, we investigated the BL2 subtype-specific TKs using the RNA-seq data from TNBC cohorts combined with clinical records [25] to identify differentially expressed (DE) TK genes and their biological functional relevance. The relationship between the TK mRNA expression levels and the disease prognosis was then analyzed, including 10-year mortality and recurrence rates. Furthermore, the effect of co-occurrence TK genes was investigated. The expression of the identified BL2-specific TK genes was validated across TNBC datasets as well as using RT-qPCR to confirm the significantly elevated expression of TK in the BL2 subtype compared to the BL1 subtype. This focused investigation contributes to a better understanding of TNBC heterogeneity, which may lead to novel clinical exploration and therapeutic applications.

## 2. Results

### 2.1. Differentially Expressed Genes and Their Functional Analysis

**Figure 1:**
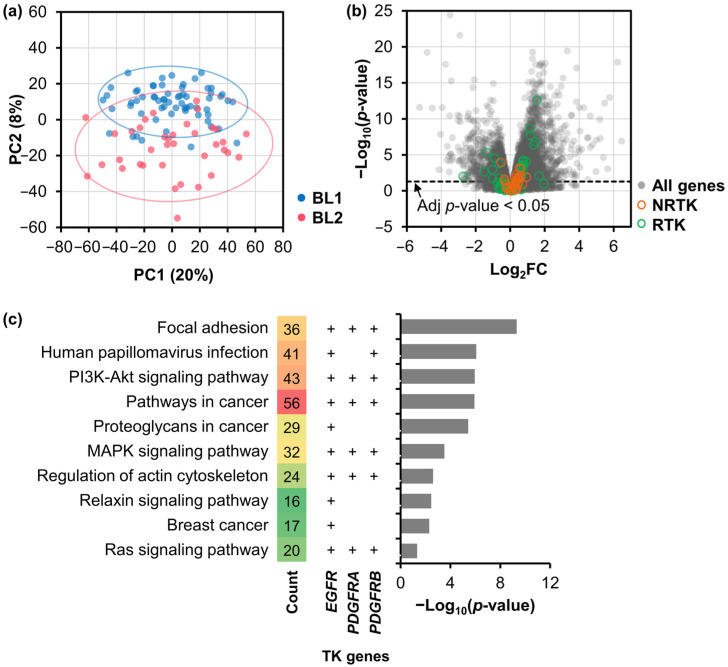
Differential gene expression analysis of the basal-like subtype of TNBC samples from the TCGA dataset. (**a**) Principal component analysis of mRNA expression in BL1 and BL2 subtypes. Each dot represents one TNBC cohort. (**b**) Volcano plot showing gene expression differences of BL2 compared to BL1. Gray dots represent genes, whereas orange and green circles represent DE genes encoding protein non-receptor (NRTKs) and receptor tyrosine kinases (RTKs), respectively. (**c**) KEGG pathway enrichment analysis for upregulated TK genes, including *EGFR*, *PDGFRA*, and/or *PDGFRB*. The enriched pathways are displayed as –Log_10_ (*p*-value), with count numbers representing the number of upregulated genes in the pathway (ranging from low in green to high in red) and a ‘+’ sign indicating the presence of a specific TK gene.

The RNA-seq data of TNBC patients from the TCGA dataset were analyzed using DESeq2 to detect DE genes in the BL2 subtype compared to the BL1 subtype [25,26]. Guided by the genomic information and the expression patterns of clinically defined ER, PR, and HER2 tumors, approximately 17% (183 cases) of TCGA breast cancer patients were diagnosed as having TNBC, with 55% being BL1 (64 cases, 35%) or BL2 (37 cases, 20%) subtypes [5]. The principal component analysis (PCA) revealed the transcriptome-level heterogeneity between these two subtypes (Figure 1a). Subsequently, BL2 subtype-specific DE genes were defined based on their significantly higher changes in gene expression compared to BL1. Overall, 1012 upregulated genes and 380 downregulated genes were differentially expressed in BL2 (Figure 1b).

To understand the biological processes and functions of the identified DE genes, Kyoto Encyclopedia of Genes and Genomes (KEGG) and Gene Ontology (GO) enrichment analysis were conducted. In the KEGG analysis, 23 pathways were enriched (Figure S1a) from upregulated DE genes. Nine pathways (39.1%) were related to human diseases, including cancer, infectious diseases, and cardiovascular disease, reflecting the multifaceted nature of these pathways. An additional seven pathways (30.4%) were related to signaling transduction and interactions. Among these, only 10 pathways associated with *EGFR*, *PDGFRA*, and/or *PDGFRB* (Figure 1c). In particular, the PI3K-Akt, MAPK, and Ras signaling pathways play pivotal roles in regulating essential cellular processes such as survival and proliferation [27,28,29]. In conjunction with focal adhesion and regulation of the actin cytoskeleton, these pathways may involve in the invasive and metastatic phenotypes of malignant cancer cells. Conversely, downregulated DE genes were significantly enriched in signaling-related pathways and human diseases (Figure S1b). Our analysis revealed a link between the PI3K-Akt signaling pathway and breast cancer in both upregulated and downregulated DE genes. This suggested intricate regulatory dynamics within these pathways, possibly involving biphasic gene regulation or context-dependent behavior. Moreover, the GO analysis showed distinct characteristics of the BL2 subtype (Figure S2). Upregulated DE genes in BL2 were associated with biological processes (BPs) related to cellular processes, developmental processes, biological regulation, and metabolic processes. Cellular components (CCs) were primarily linked to extracellular matrix and membrane-associated structures, while molecular functions (MFs) included structural and catalytic activities, as well as various binding functions.

### 2.2. Signature TK Genes of the BL2 TNBC Subtype

**Figure 2:**
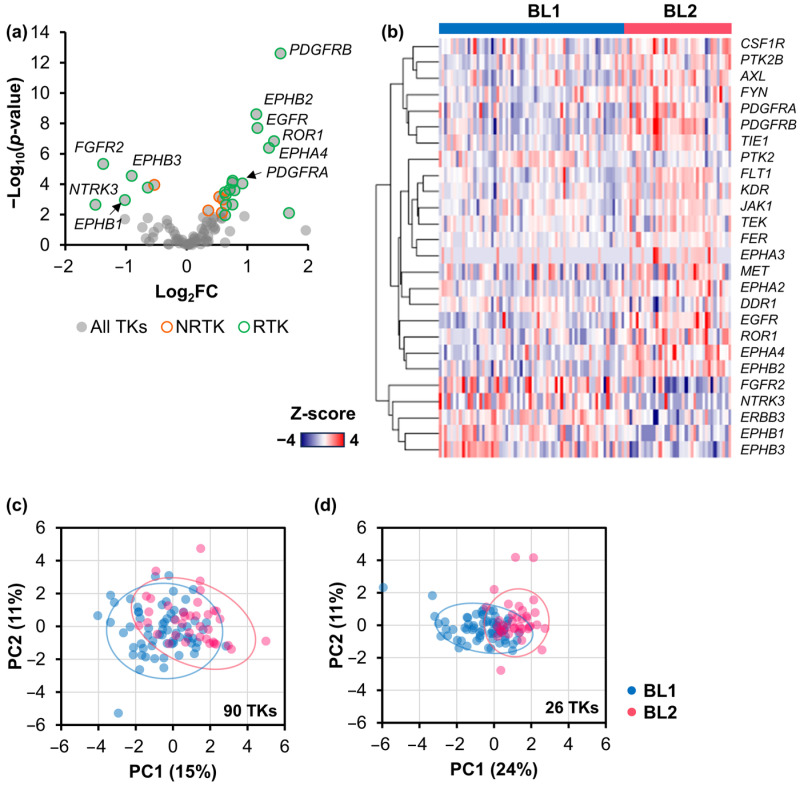
Differentially expressed TK genes in the BL2 TNBC subtype. (**a**) Volcano plot showing all TK gene expressions. Gray dots represent all DE TK genes, whereas orange and green represent non-receptor and receptor tyrosine kinase genes (NRTK and RTK, respectively). (**b**) Heatmap of the mRNA expression levels (z-score) of 26 DE TK genes within BL1 and BL2 patients. Red indicates overexpression, while blue indicates underexpression. (**c**,**d**) Principal component analysis of mRNA expression from the BL1 and BL2 TNBC subtypes where the dataset consisted of (**c**) all 90 TK genes and (**d**) only identified 26 TK genes. Each dot represents one TNBC cohort.

Identifying a distinct TK specific to the BL2 TNBC subtype could serve as a biomarker for diagnosis and potential drug target. Differential gene expression analysis revealed 26 TKs, including 5 NRTKs and 21 RTKs, that were differentially expressed in BL2 (Figure 2a), and their mRNA expression levels were displayed (Figure 2b). We then performed PCA based on all 90 TK genes (Figure 2c) and identified 26 TK genes (Figure 2d). The PCA plots showed that these identified 26 TK genes can separate the BL1 and BL2 patients using hierarchical clustering with 77% accuracy, surpassing the accuracy of 62% obtained with all 90 TK genes. This suggested the potential of using the set of identified kinases to differentiate the BL2 from the BL1 subtype. To explore the importance of these kinases, six upregulated TK genes, consisting of *EGFR*, *EPHA4*, *EPHB2*, *PDGFRA*, *PDGFRB*, and receptor tyrosine kinase-like orphan receptor 1 (*ROR1*), and four downregulated TK genes, including *EPHB1*, *EPHB3*, *FGFR2*, and the neurotrophic tyrosine receptor kinase 3 (*NTRK3*), were selected for further analysis.

### 2.3. Mortality and Recurrence Rates of BL2 TNBC Patients Expressing Signature TK Genes

**Figure 3:**
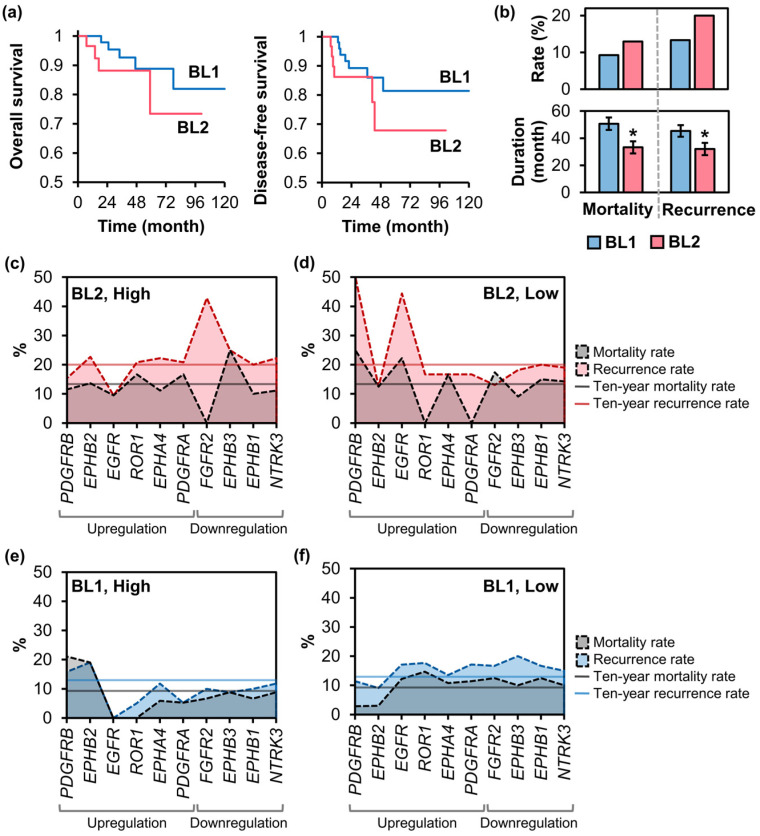
Ten-year mortality and recurrence rates of basal-like TNBC patients. (**a**) Kaplan–Meier survival curves of BL1 and BL2 TNBC patients. The curve illustrates the probability of survival (**left**), and disease-free survival over time (**right**). (**b**) (**top**) Ten-year mortality and recurrence rates of BL1 and BL2 TNBC patients from the TCGA dataset. (**bottom**) Average months of survival and recurrence-free durations. (**c**,**d**) Mortality and recurrence rates of BL2 patients with (**c**) high and (**d**) low mRNA expression levels of upregulated and downregulated TK genes. (**e**,**f**) Mortality and recurrence rates of BL1 patients with (**e**) high and (**f**) low mRNA expression levels of upregulated or downregulated TK genes. The subgroups of ‘high’ and ‘low’ were determined by mRNA expression of DE TK genes (positive and negative z-score values, respectively). Data in (**b**, **bottom**) represent mean ± standard error. The *p*-value was calculated using Student’s *t*-test: * *p* < 0.05.

The mRNA expression levels of six upregulated TK genes were analyzed in relation to disease prognosis (Figure 3a), including overall survival (10-year mortality rates) and disease-free survival (recurrence rates). According to the TCGA dataset, the BL2 subtype exhibited a higher 10-year mortality rate (13.3%) and recurrence rate (20.0%) compared to the BL1 subtype (9.3% and 13.0%, respectively) (Figure 3b, top). Furthermore, BL1 patients showed longer survival and recurrence-free duration (50.6 and 45.3 months) compared to BL2 patients (33.2 and 32.0 months) (Figure 3b, bottom), emphasizing the poor prognosis of the BL2 compared to the BL1 TNBC subtype.

To explore the link between mRNA expression and disease prognosis, BL1 and BL2 patients were categorized into ‘high’ and ‘low’ expression subgroups based on the mRNA expression levels of DE TK genes (positive and negative z-score values, respectively). Overall, a heightened mortality risk was found associated with an increased likelihood of recurrence. For example, elevated *ROR1* or *PDGFRA* expression in BL2 patients was associated with increased mortality rates (Figure 3c), while those with low expression showed improved survival and lower recurrence rates (Figure 3d). In addition, the majority of BL2 patients expressing significant amounts of TKs had high recurrence rates, except for *PDGFRB* or *EGFR*. Low expression of these TKs was associated with increased disease aggressiveness, indicated by elevated mortality and recurrence rates in BL2 patients expressing low levels of *PDGFRB* or *EGFR* (Figure 3d). In BL1 patients (Figure 3e), high expression of *PDGFRB* or *EPHB2* showed a correlation with higher mortality and recurrence rates. Furthermore, lower *EGFR*, *ROR1*, or *PDGFRA* expression levels were associated with increased disease aggressiveness in BL1 (Figure 3f). For downregulated TK genes (Figure 3c,d), all BL2 patients with high *FGFR2* expression survived in a 10-year period; however, more than 40% experienced recurrence. Considering the level of downregulated TK genes in BL1 patients, we found no noticeable relation between the mRNA expression levels and mortality/recurrence rates (Figure 3e,f). Surprisingly, the *EGFR* expression level showed a negative correlation with mortality and recurrence rates in both BL1 and BL2 patients.

### 2.4. The Effect of the Co-Occurence of TK Gene Expression on Mortality and Recurrence Rates

Due to the complexity and heterogeneity of TNBC, targeting multiple kinases would be useful for predicting prognosis that could potentially improve therapeutic outcomes [30]. To study the effects of TK expression levels on disease prognosis, only dual TK expression was analyzed (number of patients shown in Figure S3).

**Figure 4:**
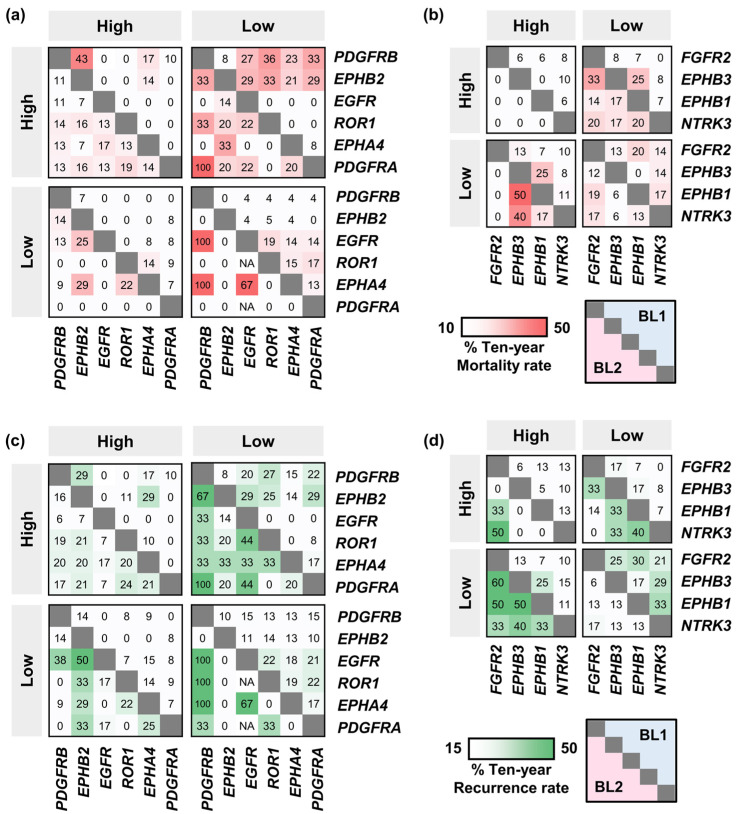
The relationship between dual expression of identified signature TK genes and TNBC prognosis within the 10-year period. The mortality rates of BL1 (**top right**) and BL2 (**bottom left**) TNBC patients with (**a**) upregulated and (**b**) downregulated TK genes. The recurrence rates of BL1 (**top right**) and BL2 (**bottom left**) TNBC patients with (**c**) upregulated or (**d**) downregulated TK genes. The 10-year mortality (red) and recurrence (green) rates are represented on the scale bar as indicated.

In BL2 patients, despite the overall elevated expression of upregulated TK pairs correlating with higher mortality (Figure 4a) and recurrence rates (Figure 4c), the contrast in mRNA expression levels of specific TK pairs correlated to higher mortality and recurrence rates than BL2′s 10-year rates, such as low *PDGFRB* paired with a high *EPHB2* or high *ROR1*, and low *EGFR* paired with a high *EPHB2* or high *ROR1*. In addition, low expression of the *EGFR*-*EPHA4* pair in BL2 resulted in 67% recurrence and mortality rates. In most cases, the mortality rate was proportional to the recurrence rate. However, patients with low *PDGFRA* expression levels showed 100% survival over the 10-year period but tended to experience recurrence regardless of gene expression levels of other upregulated TKs. The majority of patients with low *PDGFRB* or high *EPHA4* expression also tended to recur. For downregulated TK genes (Figure 4b,d), BL2 patients with high *FGFR2* expression but low *EPHB1* or *EPHB3* expression had a high recurrence rate of up to 60% with 100% survival. Furthermore, distinct kinase pairings influenced the prognosis in BL1 patients, highlighting the prognostic significance of kinase interactions.

Dual expression patterns of both upregulated and downregulated TK genes could serve as prognostic predictors (Figures S4 and S5). In BL2, high expression of both *FGFR2* and other upregulated TK genes correlated with an overall high recurrence rate, while patients with this pattern tended to survive well, with over 25% experiencing recurrence. Conversely, low expression of *FGFR2* paired with high expression of upregulated TK genes was associated with reduced recurrence but a higher mortality rate. Therefore, *FGFR2* could be a promising recurrence predictor for BL2. Furthermore, low expression of *PDGFRB* or *EGFR*, combined with low expression of *FGFR2*, *EPHB1*, or *NTRK3*, was associated with poor prognosis. In the case of BL1, high expression of either *PDGFRB* or *EPHB2* paired with low expression of downregulated TKs resulted in an overall increase in both mortality and recurrence rates. Our heatmaps displayed the relationship between different levels of TK gene expression and disease prognosis, including 10-year mortality and recurrence rates that could be useful to predict the prognosis of an individual patient.

To assess the statistical significance of our findings, Fisher’s exact test was performed. The results revealed that the mRNA expression levels of *EGFR* paired with other identified TK genes were significantly associated with disease recurrence in BL2 patients (Table 1). In contrast, the mRNA expression levels of either *PDGFRB* or *EPHB2* paired with other identified TK genes were observed to be significantly related to mortality rates in BL1 patients (Table S1).

### 2.5. Validation of Signature TK Genes with TNBC Cohorts

**Figure 5:**
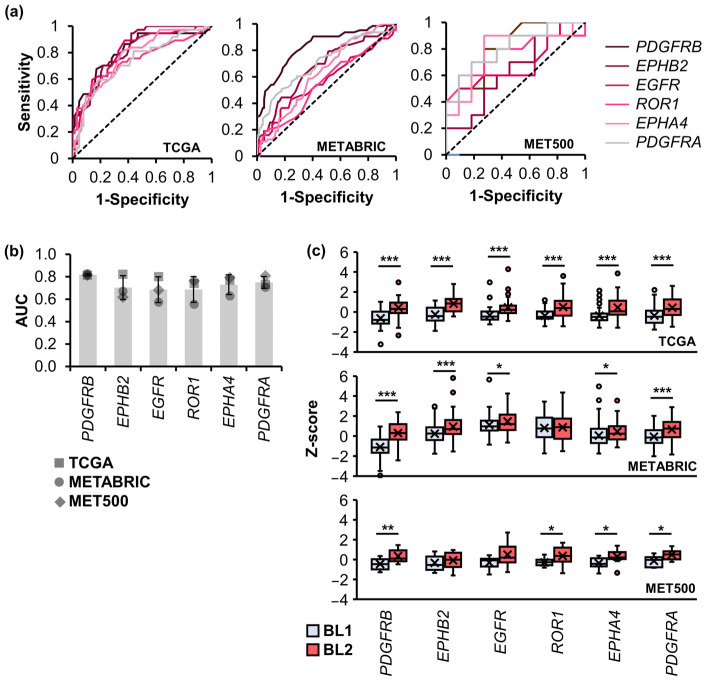
ROC analysis of identified signature TK genes. (**a**) ROC curves of six upregulated TKs plotted in a variety of datasets, including TCGA, METABRIC, and MET500. (**b**) The mean ± standard deviation AUC across the three datasets. Each symbol indicates the dataset. (**c**) The z-score of mRNA expression of upregulated TK genes from TNBC datasets, including TCGA (**top**), METABRIC (**middle**), and MET500 (**bottom**). The asterisks represent significant levels (Student’s *t*-test), * *p* < 0.05, ** *p* < 0.01, and *** *p* < 0.001.

To validate the identified BL2-specific TK genes, we performed a comparative analysis using RNA expression data from TGCA and two additional TNBC datasets, including The Molecular Taxonomy of Breast Cancer International Consortium (METABRIC) (119 BL1 and 63 BL2 patients) [31] and MET500 (11 BL1 and 10 BL2 patients) [32]. The Clinical Proteomic Tumor Analysis Consortium (CPTAC) dataset was initially considered; however, it was excluded due to limited cases (11 BL1 and 3 BL2 patients, see Figure S6a) [33]. Receiver operating characteristic (ROC) curve analysis (Figure 5a) and its area under the curve (AUC) were utilized to assess the TK gene’s performance in differentiating the BL1 and BL2 TNBC subtypes (Figure 5b). The AUC values for the upregulated TK genes in BL2 consistently exceeded 0.600, with *PDGFRB* exhibiting the highest average AUC (0.819 ± 0.008), followed by *PDGFRA* (0.750 ± 0.053). *EGFR* showed a high AUC in the TCGA and CPTAC datasets but low in the RNA microarray profiling data from the METABRIC dataset. For downregulated TK genes (Figure S6b,c), *EPHB1* and *EPHB3* had average AUC values of 0.723 ± 0.024 and 0.788 ± 0.108, respectively, while *FGFR2* and *NRTK3* displayed slightly lower AUC values. In conclusion, four BL2-specific TK candidates, including *PDGFRB*, *PDGFRA*, *EPHA4*, and *EPHB2*, have the potential to serve as biomarkers for classifying the BL2 from the BL1 TNBC subtypes with the average AUC > 0.700.

The mRNA expression (z-score) for upregulated TK genes in BL2 patients was compared to that in BL1 patients (Figure 5c). In both TCGA and METABRIC datasets, the mRNA expression of all upregulated TK genes was significantly higher in BL2 patients, except for *ROR1* in the METRABRIC dataset. In MET500, *EPHB2* and *EGFR* were the only two TKs where expression levels were not statistically significantly different between BL1 and BL2 patients. Notably, *EGFR* exhibited high variability among BL2 patients in the MET500 dataset.

### 2.6. RT-qPCR Validation of Signature Upregulated TK Genes in TNBC Cell Lines

In addition to validating the expression of identified TK genes across TNBC datasets, the representative BL1 and BL2 cell lines were selected for gene expression quantification. HCC2157 was chosen as the representative of BL1, while HCC70 and HCC1806 were selected as representatives of BL2 due to their stability of gene expression signatures derived from the TNBC subtypes [2,34,35]. HCC1187 was also included as another BL2-subtype representative due to its consistent gene expression with the BL2 subtype in both in vitro and in vivo xenograft analyses, although it was typically classified as the immunomodulatory (IM) subtype [34].

**Figure 6:**
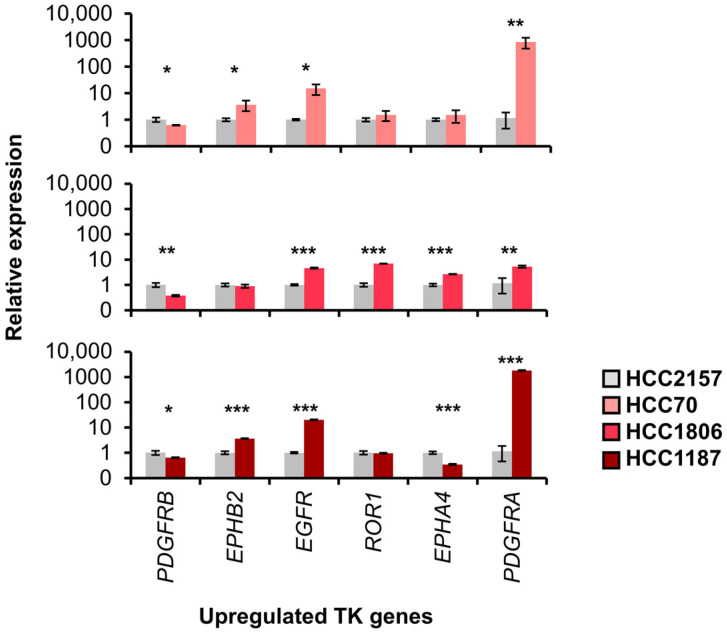
RT-qPCR analysis of relative expression of TK genes specific to the BL2 TNBC subtype. The relative gene expression values of identified upregulated TK genes in BL2 cell lines (HCC70, HCC1806, and HCC1187) were compared to those of the control BL1 cell line (HCC2157). Data represent mean, with error bars showing the standard error of three biological replicates. The asterisks denote significant level (Student’s *t*-test), * *p* < 0.05, ** *p* < 0.01, and *** *p* < 0.001.

The RNAs from each cell line were extracted to quantify the mRNA expression levels of upregulated TK genes that were identified from TCGA datasets, including *PDGFRB*, *EPHB2*, *EGFR*, *ROR1*, *EPHA4*, and *PDGFRA* (Figure 6). Among the top upregulated TK genes, *EPHB2*, *EGFR*, and *PDGFRA* showed overall consistent expression differences with the RNA-seq data. A comparison of mRNA expression revealed that *PDGFRA* and *EGFR* significantly showed the highest fold change in BL2 compared to BL1 TNBC cell lines. However, the relative mRNA expression of *PDGFRB* was lower in all BL2 cell lines, apart from high Log2FC and significance of *PDGFRB* analyzed from the TCGA RNA-seq analysis. Thus, our result from the cell line model suggested that *PDGFRA* and *EGFR* might be potential candidates for classifying BL1 and BL2 TNBC subtypes. In addition, our findings unveiled inconsistencies in TK gene expression among the BL2 cell lines. For instance, *EPHA4*, *EPHB2*, and *ROR1* from most of the BL2 cell lines were observed with slightly higher expression compared to the BL1 cell line. This indicated the heterogeneity of TNBC, especially in the BL2, which reported instability among the TNBC subtypes [36].

## 3. Discussion

TNBC is an aggressive breast cancer subtype with high invasiveness, metastatic potential, and an overall poor prognosis, with the BL2 subtype exhibiting a worse prognosis among TNBC subtypes [2,6,7]. Due to its high heterogeneity and the current lack of valid targeted treatment, along with the limited efficacy of chemotherapy, which is the primary treatment for TNBC, targeted therapeutic strategies based on specific molecules and signaling pathways in TNBC hold promise for more effective treatment [2,37,38]. In this study, uniquely expressed TKs in the BL2 subtype were identified as potential prognostic markers and therapeutic targets. Our findings from transcriptomic analysis were then validated by cross-referencing with other datasets of TNBC cohorts and gene expression from cell lines using RT-qPCR. The crucial information about the TKs and their relations to prognosis was revealed, which could lead to a pivotal tool for accurate diagnosis and effective therapeutic strategies for the BL2 TNBC subtype.

Despite the similarity among basal-like subtypes of TNBC, BL1 and BL2 subtypes have distinct gene expression and pathway alterations. Several growth factor receptors were distinctly upregulated in BL2, such as EGFR (encoded by *EGFR*), PDGFRα (encoded by *PDGFRA*), and PDGFRβ (encoded by *PDGFRB*), making these receptors potentially candidates as diagnostic markers or targeted therapy for the BL2 TNBC subtype. In particular, EGFR has been shown to be overexpressed in 70–78% of basal-like TNBC compared to non-TNBC, and its related pathway, such as the EGFR/MAPK signaling, was found to be more active in the BL2 subtype [5,22,35]. However, our results suggested that using EGFR as the BL2-specific marker may be ineffective due to its expression inconsistency in the classification performance between BL1 and BL2 subtypes across TNBC datasets. PDGFRα and PDGFRβ, on the other hand, could serve as the potential BL2-specific markers with high sensitivity and specificity (AUC > 0.7 in all three validation datasets), although PDGFRα and PDGFRβ have been previously reported to be associated with basal-B like cell lines [39] and identified as a biomarker for the mesenchymal TNBC [40], respectively. As a result, our identified upregulated TKs, such as PDGFRα and PDGFRβ, could serve as potential markers for differentiating the BL2 from the BL1 subtype and therapeutic targets.

Our findings also suggested potential interactions among *EGFR*, *PDGFRA*, and *PDGFRB* genes in TNBC (Figure S7), as they were found involved in the PI3K-Akt, MAPK, and Ras signaling pathways, identified through KEGG pathway enrichment analysis. This observation aligns with a previous study indicating that the loss of the TNBC tumor suppressor protein, PTPN12, leads to the activation of EGFR and PDGFRβ pathways [41,42]. EGFR activation can stimulate the PI3K-Akt and MAPK pathways, contributing to cell survival and proliferation [43,44]. Meanwhile, activated PDGFRα and PDGFRβ also engage in these pathways, further enhancing cell growth and angiogenesis. The abnormal expression level of these TKs, especially EGFR, was reported to affect MAPK and Ras signaling in TNBC [29]. In addition to upregulated TKs, our finding revealed the importance of downregulated TKs, i.e., FGFR2 (encoded by *FGFR2*), associating with the PI3k-Akt signaling pathway. Dysregulated PI3K-Akt signaling is a common oncogenic aberration of TNBC, driving increased cell proliferation, survival, and invasion, thus contributing to tumor progression [5,27,45]. The PI3K-Akt pathway has emerged as a promising therapeutic approach for TNBC, which currently is under clinical investigation [38,46]. The collaborative signaling among EGFR, PDGFRα, PDGFRβ, and FGFR2 proteins could result in a synergistic effect on promoting tumor progression making them valuable targets for cancer therapy. However, more investigation is needed to elucidate the interplay among these kinases in TNBC.

The mRNA expression levels of TKs in the BL2 subtype exhibited significant associations with 10-year mortality and recurrence rates. Despite the typically observed *EGFR* overexpression in TNBC, our results revealed that BL2 patients with low *EGFR* expression tended to survive (mortality rate of 22%) and recover (recurrence rate of 44%) worse than those with high *EGFR* expression. This observation aligns with previous reports highlighting that low *EGFR* expression was found in metastatic cells despite its overexpression in the primary tumor [36,47]. Similarly, low *PDGFRB* expression was found to be associated with high mortality and recurrence rates in BL2 patients (25% and 50%, respectively). Hence, the expression levels of *EGFR* or *PDGFRB* may differently affect the disease prognosis, which could explain the limited efficacy of EGFR tyrosine kinase inhibitors (TKIs) in clinical trials [22,48] and the unexpected outcomes of TKIs targeting PDGFRβ in TNBC treatment [40,49]. Unexpectedly, high expression of downregulated *FGFR2* was linked to a high recurrence rate in the BL2 patients, although all patients survived. This suggested the possibility of using *FGFR2* as a disease progression predictor for BL2. Still, the role of FGFR2 in TNBC appears to be multidimensional, since it has been linked to varying survival rates in different studies [50,51]. These findings highlight the importance of accurately identifying TNBC subtypes for effective treatment. Thus, *EGFR*, *PDGFRB*, and *FGFR2* could be used as prognosis predictors for the BL2 subtype. In addition to the mRNA expression of TK genes, DNA mutations were found to be involved in disease prognosis that lower mutation levels correlate with worse progression-free intervals [5,52]. This is consistent with our findings that BL2 patients have a lower DNA mutation rate (Figure S8 and Table S2).

As biological systems work in concert, relying on a single kinase as a marker would be insufficient; therefore, targeting multiple kinases may enhance the reliability of TNBC subtype diagnosis. While our findings revealed novel associations between mRNA expression levels and disease progression, these relationships have not been reported in TNBC. For instance, poor prognosis, especially the recurrence of disease, was mostly found in BL2 patients with low *EGFR* expression paired with specific TKs. The co-expression of EGFR and FGFR2 linked to resistance to EGFR kinase inhibitors in esophageal cancer was reported [47], while the co-expression of EGFR and EPHA2 was found associated with the promotion of tumorigenesis in lung and colorectal cancer [53]. Despite the structural similarity between the kinase domains of EPHA4 and EPHA2, a bispecific anti-EGFR/EPHA2 antibody demonstrated superior efficacy in suppressing aggressive TNBC and pancreatic cancer tumors in xenograft models compared to the anti-EGFR antibody cetuximab alone [54]. Although targeting multiple signaling pathways with TKIs shows promise in cancer treatment, the increased risk of toxicities was observed with the inhibition of multiple kinases [55,56,57]. Enhancing TKI selectivity for specific targets is crucial for maintaining effectiveness and safety [55,56]. Although the patient cohort in this prognosis study included 84 patients, the dual expression analysis of TK pairs was limited by the number of patients. In certain instances, the analysis was limited to cases with fewer than three patients (Figure S3); therefore, the results of dual-TK expression analysis should be interpreted with caution. A larger dataset is recommended for further investigation.

The RT-qPCR was employed to validate the expression of identified kinases within a TNBC cell line model. HCC70, HCC1806, and HCC1187 were selected as representative cell lines for the BL2 subtype, and HCC2157 for the BL1 subtype [2,34,35]. Notably, among the upregulated TK genes, *EGFR* and *PDGFRA* consistently displayed high expression in all BL2 cell lines, confirming their potential as BL2-specific markers, but not for *PDGFRB*. The observed discrepancy of *PDGFRB* expression between RT-qPCR results and RNA-expression analyses across TNBC datasets may arise from multiple factors, such as in vitro culture conditions and the impact of the microenvironment [58] where PDGFRβ is abundantly expressed [59,60,61]. Even though cell lines are not an optimal model for translational research validation, cell models, such as organoids or patient-derived xenografts, can improve research repeatability and relevance [62,63]. Our study presents a valuable choice for utilizing cell lines that better represent the BL2 TNBC subtype. Thus, understanding the mechanisms of tyrosine kinases and their associated pathways not only expands therapeutic options but also serves as a diagnostic marker, enables accurate prognosis prediction, and improves the treatment efficacy of TNBC.

## 4. Materials and Methods

### 4.1. RNA-Seq Datasets

The transcripts per million (TPM) RNA-seq data of TNBC patients were chosen from the TCGA-BRCA project [25] (UCSC XENA Project—https://xenabrowser.net/, accessed on 17 July 2022). A total of 183 TNBC tumor samples were selected and categorized into BL1 (64 samples), BL2 (37 samples), LAR (27 samples), and M (55 samples) TNBC subtypes [5]. Only RNA-seq data of BL1 and BL2 subtypes (total of 101 samples, excluding long noncoding RNAs) were included for differential gene expression analysis. Patient information, including age, tumor stage, and treatment record, are summarized in Table S3.

### 4.2. Gene Expression Analysis

Differential gene expression analysis of RNA-seq data was conducted using DESeq2 (v.1.30.1) [26] via R (v4.0., R Foundation for Statistical Computing). The list of identified genes were differentially expressed in BL2 compared to BL1. To visually represent the findings, a volcano plot was created, illustrating the relationships between log_2_ fold change (log_2_FC) and −log_10_ (*p*-value) of each gene. Only the genes with adjusted *p*-value < 0.05 were considered statistically significant, and their expression was quantified based on log_2_FC, which can categorize them as upregulated (log_2_FC ≥ 0.9) or downregulated (log_2_FC ≤ −0.9). Only the TK genes were used in further analysis.

### 4.3. Gene Ontology and KEGG Pathway Enrichment Analysis

To further investigate the biological functions of the identified DE genes, all upregulated or downregulated DE genes were submitted in the web-based bioinformatics tool, the Database for Annotation, Visualization, and Integrated Discovery (DAVID, https://david.ncifcrf.gov/, accessed on 21 September 2023) [64,65]. The analysis utilized the functional annotation tool, with a focus on GO enrichment analysis and KEGG pathway analysis. The default parameters with medium stringency were implemented and computed over the background of the whole human genome. The results with *p*-value < 0.05 and a percentage count of more than 1.5 were selected.

### 4.4. Gene Expression Validation

Additional transcriptomic datasets of TNBC patients were employed to validate our findings. METABRIC dataset [31] includes normalized RNA microarray profiles from 1981 fresh-frozen primary breast cancer specimens, comprising 182 samples designated as BL1 (119 samples), and BL2 (63 samples) TNBC subtypes [5]. The MET500 dataset [32] contains gene expression profiles from 500 patients with metastatic cancers originating from over 30 primary sites and biopsied from more than 22 organs, with 21 breast cancer samples classified as BL1 (11 samples), and BL2 (10 samples) [5]. CPTAC dataset [33] includes proteomic data obtained through mass spectrometry, along with next-generation DNA and RNA sequencing data from 122 primary breast cancer patients without any prior treatment. This dataset included 14 samples classified into BL1 (11 samples), and BL2 (3 samples) TNBC subtypes [5].

To validate the gene expression across datasets, the z-score of identified TK gene expression was compared using the ROC curve to evaluate the sensitivity and specificity of identified markers in classifying the BL1 and BL2 TNBC subtype. In addition, AUC was used to represent the overall performance of identified TK genes as signature genes to classify TNBC subtypes. In addition, the mRNA expression of each identified TK gene was compared between BL1 and BL2 patients within the same dataset.

### 4.5. Mortality and Recurrence Rates

Assessing the disease prognosis is essential for guiding treatment decisions. To evaluate the significance of the BL2-specific TK genes, we analyzed their association with the 10-year mortality rate (referring to the percentage of individuals who did not survive the disease) and the 10-year recurrence rate (referring to the rate of patients with TNBC returning after initial treatment). This analysis contained a total of the top ten TK genes, including six upregulated TKs and four downregulated TKs. The study involved a cohort of 84 cases, comprising 54 BL1 and 30 BL2 TNBC patients from the TCGA dataset, after the exclusion of cases with incomplete data.

The mortality and recurrence rates were defined as the ratio of patients who failed to survive and those who experienced a recurrence to the total number of patients within 10 years, starting from the diagnostic date. Notably, data for patients with survival or recurrence times exceeding 10 years were truncated at the 10-year mark to ensure a consistent and standardized analysis.

### 4.6. Classification of Patients with High or Low TK Gene Expression

BL1 and BL2 TNBC patients from the TCGA dataset were categorized into a ‘high’ or ‘low’ group based on the mRNA expression levels of a specific TK gene. This classification was determined by the z-score of mRNA expression, where ‘high’ expression was indicated by a positive z-score (indicating a higher expression level than the average among BL1 and BL2 patients), and ‘low’ expression was indicated by a negative z-score (indicating a lower expression level than the average among BL1 and BL2 patients). For dual TK expression, the number of patients with dual TK expression is displayed in Figure S3. To assess the statistical significance of our findings, Fisher’s exact test was performed.

### 4.7. RT-qPCR

BL1 (i.e., HCC2157) and BL2 (i.e., HCC70, HCC1187, and HCC1806) cell lines, purchased from the American Type Culture Collection (ATCC), were used as the representatives of disease. They were cultured in Roswell Park Memorial Institute Medium (RPMI 1640) (Thermo Fisher Scientific, Waltham, MA, USA) with 10% FBS and 1% Penicillin/Streptomycin at 37 °C in a humidified 5% CO_2_ incubator. Each cell line was cultured in three replicates for RNA extraction using the RNeasy Mini Kit (Qiagen, Hilden, Germany). The quantification assessment of the extracted RNA was performed using the NanoDrop 2000 spectrophotometer (Thermo Fisher Scientific, Waltham, MA, USA). The extracted RNA underwent reverse transcription following the iScript™ Reverse Transcription Supermix kit (Bio-Rad Laboratories, Hercules, CA, USA) using random hexamers. Quantitative PCR (qPCR) was performed using iTaq™ Universal SYBR^®^ Green Supermix (Bio-Rad Laboratories, Hercules, CA, USA). The qPCR for each sample was performed in technical triplicate. The mRNA expression levels were normalized to GAPDH, and the normalized expression of triplicates was then averaged. Relative quantification of mRNA expression in TK genes within the BL2 cell line was calculated using the BL1 cell line as a control.

### 4.8. Statistical Analysis

All data analyses were performed in R. The statistical analyses were performed in MATLAB (R2023a, MathWorks, Natick, MA, USA). The statistical details of all experiments are reported within the text and figure legends.

## 5. Conclusions

The aggressive nature of TNBC, especially the BL2 subtype, presents a significant clinical challenge due to its heterogeneity and the absence of clear treatment targets. Combining transcriptome analysis, experimental RT-qPCR validation, and additional TNBC datasets uncovered potential BL2-specific tyrosine kinase as identifiers to differentiate between TNBC subtype BL1 and BL2, including PDGFRα and PDGFRβ, leading to therapeutic targets and TNBC diagnostic biomarker. Furthermore, dual expression of EGFR with several identified TKs, such as EPHA4, FGFR2, or PDGFRα, could potentially serve as disease prognostic markers. These findings offer a promising avenue for precise diagnosis and targeted therapy, addressing a critical need for TKs target for BL2 TNBC treatment.

## Figures and Tables

**Table 1:** The significant association between the level of TK gene expression and the prognosis in BL2 patients.

Condition 1	Condition 2	*p*-Value	
		Mortality	Recurrence
*FGFR2* ^H^/*EPHB3* ^L^	*FGFR2* ^L^/*EPHB3* ^L^	ns	0.024
*EGFR* ^H^/*NTRK3* ^L^	*EGFR* ^L^/*NTRK3* ^L^	ns	0.028
*EGFR* ^H^/*EPHB1* ^L^	*EGFR* ^L^/*EPHB1* ^L^	ns	0.032
*EGFR* ^H^/*EPHB2* ^H^	*EGFR* ^L^/*EPHB2* ^H^	ns	0.039
*EGFR* ^H^/*EPHA4* ^L^	*EGFR* ^L^/*EPHA4* ^L^	0.045	0.045
*EGFR* ^H^/*FGFR2* ^L^	*EGFR* ^L^/*FGFR2* ^H^	ns	0.046
*EGFR* ^H^/*ROR1* ^H^	*EGFR* ^L^/*ROR1* ^H^	ns	0.047
*EGFR* ^H^/*PDGFRA* ^H^	*EGFR* ^L^/*PDGFRA* ^H^	ns	0.047

The superscript ‘^H^’ or ‘^L^’ denotes high or low expression, respectively. ‘ns’ indicates not significant (*p*-value > 0.05) where the *p*-value was calculated using Fisher’s exact test.

## Data Availability

TCGA RNA-seq gene expression data were available through https://xenabrowser.net/ (accessed on 17 July 2022) (cohort: TCGA Pan-Cancer (PANCAN); dataset ID: tcga_RSEM_gene_tpm). CPTAC mRNA-seq z-score was available through https://www.cbioportal.org/datasets (accessed on 15 December 2022) (Breast Cancer (Proteogenomic landscape of breast cancer (CPTAC, Cell 2020) [33]). METABRIC mRNA microarray z-score was available through https://www.cbioportal.org/datasets (accessed on 25 September 2022) (Breast Cancer (METABRIC, Nature 2012 & Nat Commun 2016) [31,67,68]). MET500 RNA-seq z-score was available through https://xenabrowser.net/ (accessed on 25 September 2022) (cohort: MET500 (expression centric) [32]; dataset ID: MET500/geneExpression/M.mx.log2.zscore.txt).

## Supplementary Materials

The following supporting information can be downloaded at: https://www.mdpi.com/article/10.3390/ijms25031405/s1. Reference [66] is cited in the Supplementary Materials.
