# Supplementary material for: Prognostic Markers in Tyrosine Kinases Specific to Basal-like 2 Subtype of Triple-Negative Breast Cancer
Source: Int J Mol Sci. 2024 Jan 24;25(3):1405. doi: 10.3390/ijms25031405 (PMC10855431; doi:10.3390/ijms25031405)
Supplement: Supplementary file 1 [file ijms-25-01405-s001.zip › ijms-2775413-supplementary.pdf]

Supplementary Material

Prognostic Markers in Tyrosine Kinases Specific to Basal-Like 2 Subtype of Triple-Negative Breast Cancer

Praopim Limsakul [1,2], Pongsakorn Choochuen [3], Thawirasm Jungrungrueang [3] and Krit Charupanit [3,*]

1 Division of Physical Science, Faculty of Science, Prince of Songkla University, Songkhla 90110, Thailand; praopim.l@psu.ac.th
2 Center of Excellence for Trace Analysis and Biosensor (TAB-CoE), Faculty of Science, Prince of Songkla University, Songkhla 90110, Thailand
3 Department of Biomedical Sciences and Biomedical Engineering, Faculty of Medicine, Prince of Songkla University, Songkhla 90110, Thailand; pongsakorn.mwit20@gmail.com (P.C.); thawirasm.j@psu.ac.th (T.J.)
* Correspondence: krit.ch@psu.ac.th

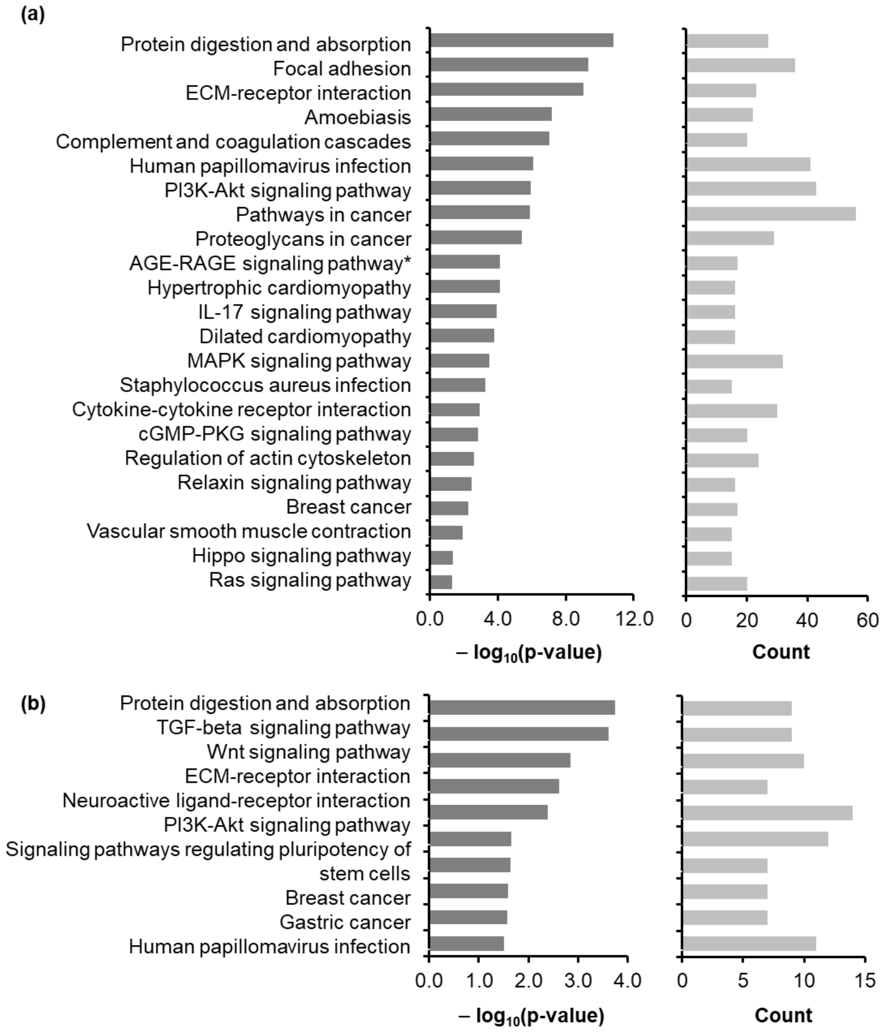

Figure S1. KEGG pathway enrichment analysis for differentially expressed genes in the BL2 subtype.

KEGG analysis for (**a**) upregulated and (**b**) downregulated DE genes. The enriched pathways were displayed as a $-\text{Log}_{10}$(*p*-value), with a count representing the number of genes in the pathway.

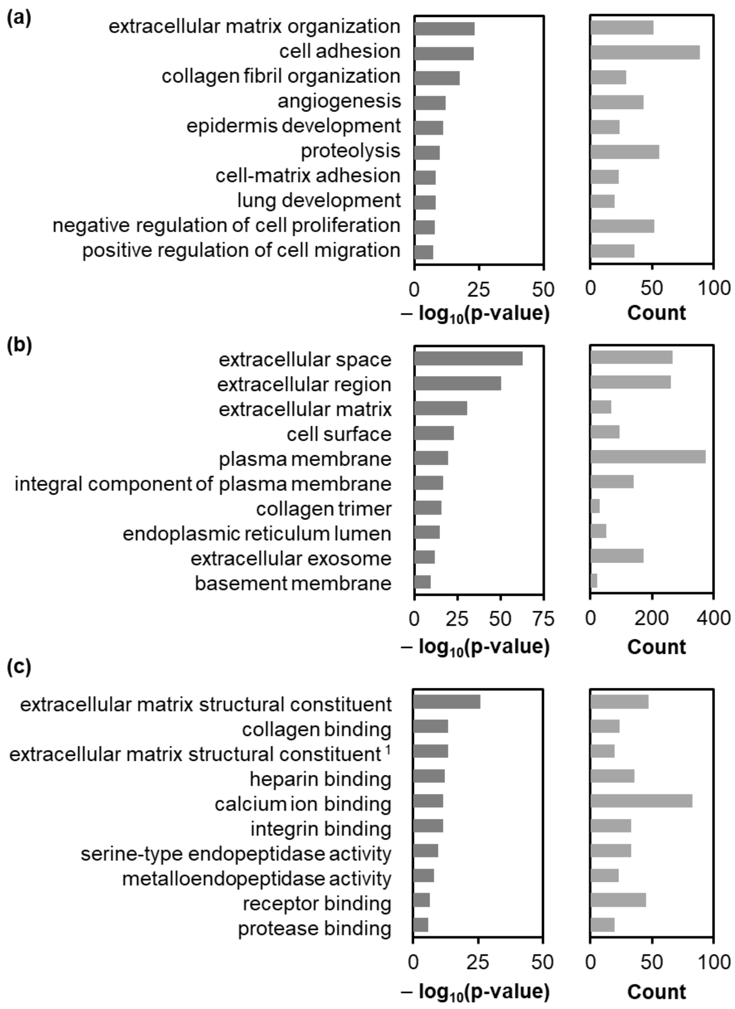

Figure S2. GO enrichment analysis of differentially expressed genes in the BL2 subtype.

GO analysis for upregulated DE genes, examining (**a**) biological processes, (**b**) cellular processes, and (**c**) molecular functions. Enriched pathways were visualized with $-\text{Log}_{10}(p\text{-value})$, along with a count indicating the number of genes within each enrichment.

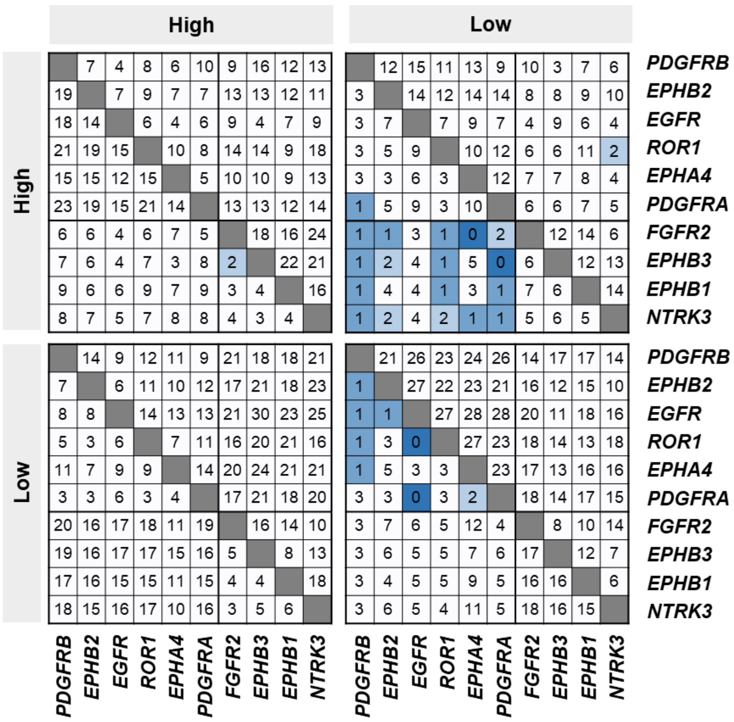

Figure S3. The relationship between dual expression of identified TK genes and the number of cohorts. The number in each point represents the number of patients.

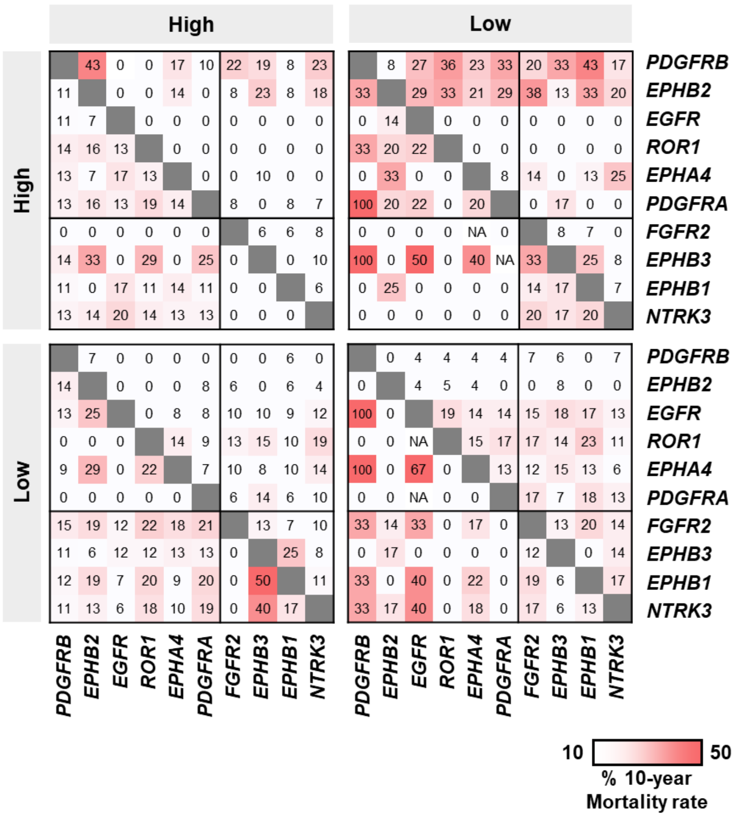

Figure S4. The relationship between dual expression of identified TK genes and the ten-year mortality rate. The scale bar represents the ten-year mortality rate, ranging from 10% (white) to 50% (red).

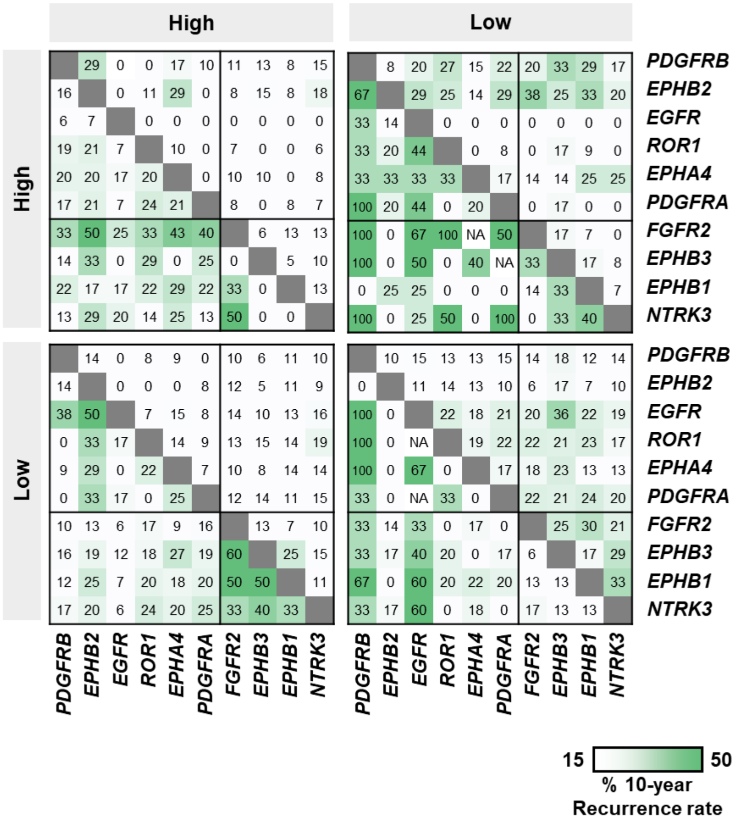

Figure S5. The relationship between dual expression of identified TK genes and the ten-year recurrence rate. The scale bar represents the ten-year recurrence rate, ranging from 15% (white) to 50% (green).

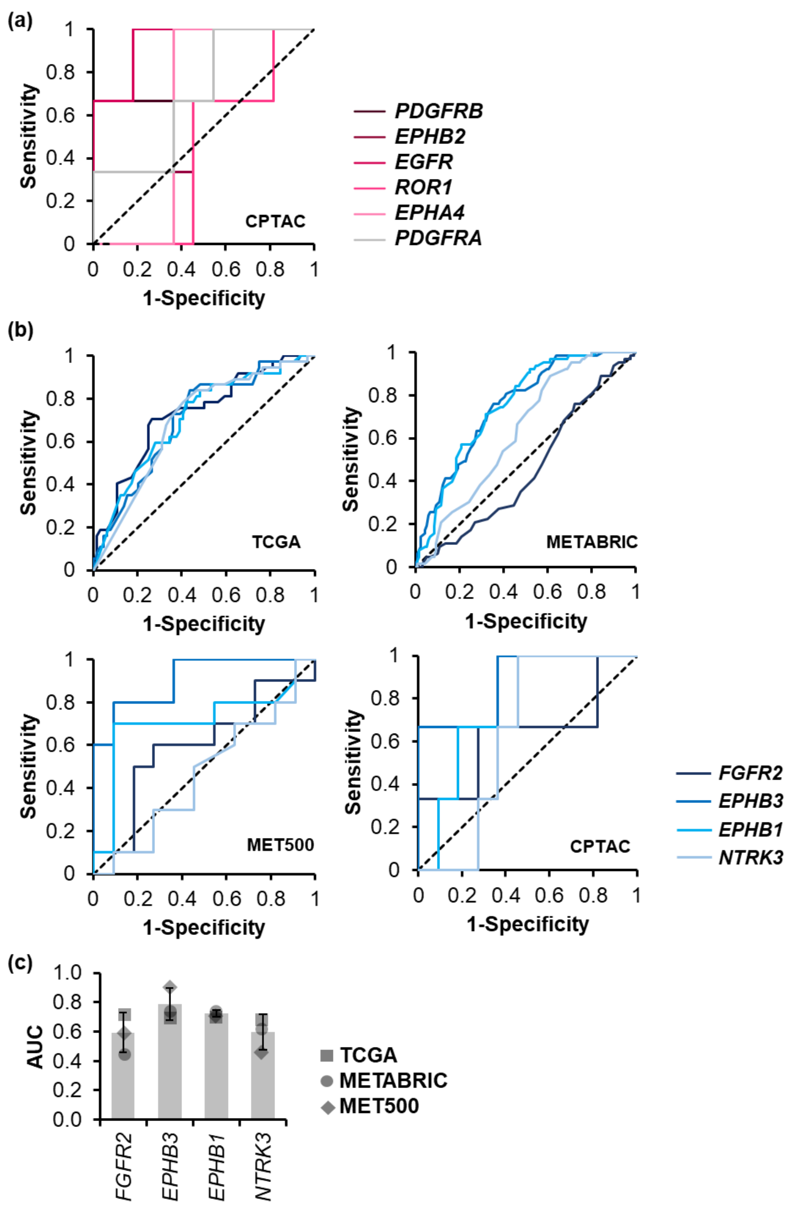

Figure S6. ROC analysis of identified TK genes across TNBC datasets of the BL1 subtype and CPTAC TNBC dataset of the BL2 subtype.

(**a**) For BL2, ROC curves of six upregulated TKs plotted in the CPTAC TNBC dataset. (**b**) For BL1, ROC curves of six upregulated TKs plotted in a variety of datasets, including TCGA, METABRIC, MET500, and CPTAC. (**c**) The average AUC across the three datasets. Each symbol indicates the datasets. Data in (c) are mean ± standard deviation.

(a)

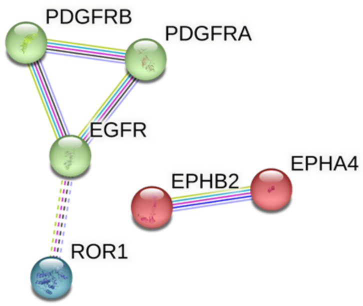

(b)

KEGG Pathways	FDR	Matching proteins
EGFR tyrosine kinase inhibitor resistance	0.003	PDGFRA, PDGFRB, EGFR
Central carbon metabolism in cancer	0.003	PDGFRA, PDGFRB, EGFR
Gap junction	0.003	PDGFRA, PDGFRB, EGFR

Reactome Pathways	FDR	Matching proteins
Constitutive Signaling by Aberrant PI3K in Cancer	0.003	PDGFRA, PDGFRB, EGFR
PI5P, PP2A and IER3 Regulate PI3K/AKT Signaling	0.0036	PDGFRA, PDGFRB, EGFR
Downstream signal transduction	0.0168	PDGFRA, PDGFRB

Figure S7. Protein-protein interaction networks of upregulated tyrosine kinases.

(a) Analysis using the Search Tool for the Retrieval of Interacting Genes/Proteins (STRING) analysis [66] reveals protein interaction networks among the upregulated tyrosine kinases (TKs): PDGFRB, EPHB2, EGFR, ROR1, EPHA4, and PDGFRA. Employing a high-confidence cut-off (0.700) and k-means clustering (number of clusters = 3), the resulting networks illustrate TKs as nodes interconnected by lines with the solid lines representing validated interactions and the dotted lines representing inter-cluster edges. (b) The functional enrichments are through the KEGG pathways and the Reactome pathways. FDR is a false discovery rate.

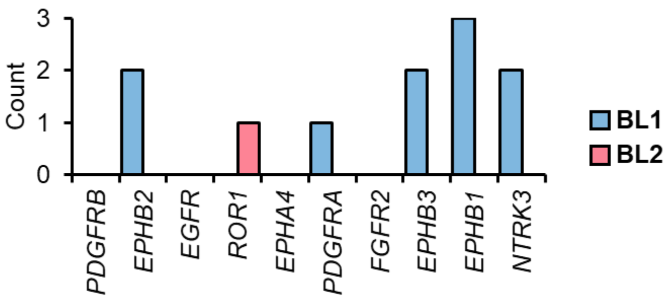

Figure S8. Mutations of identified tyrosine kinases found in BL1 or BL2 patients.

The bar graph illustrates the mutation observed in the identified TK genes along with the corresponding patient count.

Table S1. The significant association between TK gene expression and the mortality/recurrence rate in BL1 patients.

Condition 1	Condition 2	*p*-value	
		Mortality	Recurrence
$PDGFRB^H/EPHB2^H$	$PDGFRB^L/EPHB2^L$	0.011	ns
$PDGFRB^H/EPHB1^L$	$PDGFRB^L/EPHB1^L$	0.017	ns
$EPHB2^H/PDGFRA^L$	$EPHB2^L/PDGFRA^L$	0.019	ns
$EPHB2^H/FGFR2^L$	$EPHB2^L/FGFR2^L$	0.028	ns
$PDGFRB^H/ROR1^L$	$PDGFRB^L/ROR1^L$	0.029	ns
$EPHB3^H/EPHB1^H$	$EPHB3^H/EPHB1^L$	0.037	ns
$PDGFRB^H/ROR1^L$	$PDGFRB^L/ROR1^H$	0.037	ns
$EPHB2^H/EGFR^L$	$EPHB2^L/EGFR^L$	0.039	ns
$EPHB2^H/EPHB1^L$	$EPHB2^L/EPHB1^L$	0.042	ns
$EPHB2^H/ROR1^L$	$EPHB2^L/ROR1^L$	0.042	ns
$PDGFRB^H/PDGFRA^L$	$PDGFRB^L/PDGFRA^L$	0.044	ns
$EPHB2^H/EPHB3^H$	$EPHB2^L/EPHB3^H$	0.048	ns
$PDGFRB^H/NTRK3^H$	$PDGFRB^L/NTRK3^H$	0.048	ns

The superscript “^{H}” or “^{L}” denotes high or low expression, respectively. “ns” indicates not significant (p-value > 0.05) where the p-value was calculated using Fisher’s exact test.

Table S2. The number of BL1 or BL2 patients with mutations and the average mutation per patient.

Mutation of	BL1 (n = 64)		BL2 (n = 37)	
	**Patient with mutation (%)**	**Average mutation (per patient)**	**Patient with mutation (%)**	**Average mutation (per patient)**
All TK genes (90 genes)	17 (26.6%)	2.24 (38 genes)	9 (24.3%)	1.11 (10 genes)

Table S3. Patient characteristics.

Characteristics	BL1	BL2
	**n (%)**	**n (%)**
**Total number of patients**	64	37
**Age (years)**		
< 30	1 (2)	0
31-40	6 (9)	2 (5)
41-50	20 (31)	10 (27)
51-60	18 (28)	11 (30)
61-70	11 (17)	8 (22)
>70	8 (13)	6 (16)
**Tumor stage**		
I	9 (14)	6 (16)
II	48 (75)	20 (54)
III	5 (8)	9 (24)
IV	0	1 (3)
NA	2 (3)	1 (3)
**Race**		
Black	24 (38)	8 (22)
White	31 (48)	27 (73)
Others	9 (14)	2 (5)
**Radiation Therapy**		
Received	31 (48)	21 (57)
Not received	25 (39)	15 (40)
NA	8 (13)	1 (3)

"NA" indicates not available.
